# A Biological Rationale for Musical Scales

**DOI:** 10.1371/journal.pone.0008144

**Published:** 2009-12-03

**Authors:** Kamraan Z. Gill, Dale Purves

**Affiliations:** Center for Cognitive Neuroscience and Department of Neurobiology, Duke University, Durham, North Carolina, United States of America; Massachusetts Institute of Technology, United States of America

## Abstract

Scales are collections of tones that divide octaves into specific intervals used to create music. Since humans can distinguish about 240 different pitches over an octave in the mid-range of hearing [1], in principle a very large number of tone combinations could have been used for this purpose. Nonetheless, compositions in Western classical, folk and popular music as well as in many other musical traditions are based on a relatively small number of scales that typically comprise only five to seven tones [2]–[6]. Why humans employ only a few of the enormous number of possible tone combinations to create music is not known. Here we show that the component intervals of the most widely used scales throughout history and across cultures are those with the greatest overall spectral similarity to a harmonic series. These findings suggest that humans prefer tone combinations that reflect the spectral characteristics of conspecific vocalizations. The analysis also highlights the spectral similarity among the scales used by different cultures.

## Introduction

**Figure 1:**
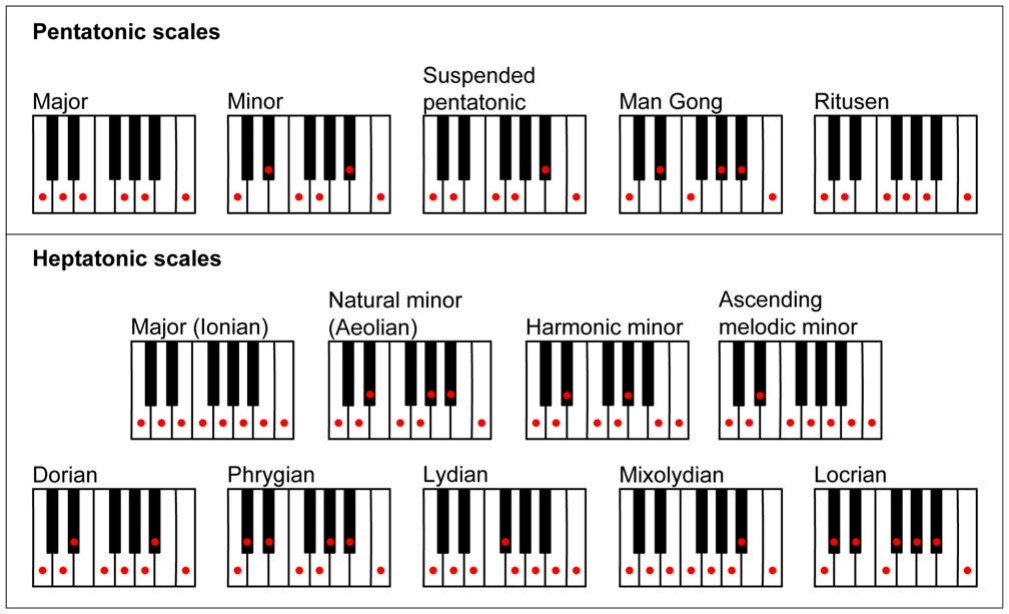
Pentatonic and heptatonic scales (included tones are indicated by red dots). The five pentatonic scales are modes of the same set of notes, the only difference being the starting note or tonic. Seven of the nine heptatonic scales shown are also modes that entail the same notes in different arrangements (the exceptions are the harmonic and melodic minor scales). There are three unique forms of the minor heptatonic scale: the natural, harmonic and melodic (the melodic minor scale shown is designated as ascending since this scale is identical to the natural minor scale when descending). Although the scales shown begin and end on specific notes of the keyboard, each could begin on any note and retain its identity as long as all intervals between notes remained the same. Scale tones are represented on keyboards for didactic purposes only in this and subsequent figures and should not be interpreted as being tuned in equal temperament (see Methods).

The most widely employed scales (also called modes) in Western music over the last few centuries have been the major and minor pentatonic and heptatonic (diatonic) scales (Figure 1). The other scales illustrated are commonly found in early liturgical music and, more recently, in folk music, modern jazz and some classical compositions [5], [7]. These same five-note and seven-note collections are also prevalent in traditional Indian, Chinese and Arabic music, although other scales are used as well [2], [3], [8]–[10]. These historical facts present an obvious puzzle: given the enormous number (billions) of possible ways to divide octaves into five to seven tonal intervals, why have only a few scales been so strongly favored?

Not surprisingly, a number of investigators have grappled with the general issue of scale structure. One approach has used consonance curves [11] to show that the consonant harmonic scale tones are defined by small integer ratios [12], [13]. This method has not, however, been used to predict any specific scale structures. A different approach to understanding scales has depended on the concept of a generative grammar in linguistics, asking whether musical patterns might define a “musical grammar” [14]. Again, this concept has not been applied to the prediction of preferred scale structures. A third approach has used error minimization algorithms to predict scale structures under the assumption of competing preferences for small integer ratios and equal intervals between successive scale tones [15], [16]. This method can account for the structure of the equal-tempered 12-tone chromatic scale but cannot account for any of the five to seven-tone scales commonly used to make music. Moreover, no basis was provided for the underlying assumptions. Other analyses have predicted scales with as many as 31 intervals, which are rarely used to make music [17], [18]. In short, none of these approaches explains the widespread human preference for a small number of particular scales comprising five to seven tones, or provides a biological rationale for this predilection.

Here we examine the possibility that the thread tying together the scales that have been preferred in music worldwide is their overall similarity to the spectral characteristics of a harmonic series. The comparison of musical intervals to a harmonic series is not new. Helmholtz [19] first proposed that the relative consonance of musical dyads derives from harmonic relationships of the two tones. More recently, Bernstein [20] suggested that scale structure is determined by the appeal of the lower harmonics that occur in naturally generated harmonic series. For example, assuming octave equivalence, the intervals between the tones of the major pentatonic scale are nearly the same as the intervals between the first nine harmonics of a harmonic series. However, a number of flaws were later pointed out in this argument [14]. For one thing, the last note of the major pentatonic scale only roughly approximates the seventh harmonic. Moreover, widely used scales containing a minor second interval are not predicted, as this interval does not occur until the 15^th^ and 16^th^ harmonics of a harmonic series.

The different approach we take here is to quantitatively compare the harmonic structure that defines each interval in a possible scale to a harmonic series, rather than to consider only the intervals between fundamental frequencies and individual harmonics. Accordingly our analysis does not depend on intervals and scales precisely mimicking a harmonic series, but evaluates degrees of similarity. The average similarity of all intervals in the scale is then used as a measure of the overall similarity of the scale under consideration to a harmonic series. In this way we assess whether the scales with the highest degree of similarity to a harmonic series are in fact the scales commonly used to make music.

## Materials and Methods

### Measurement of scale similarity to a harmonic series

**Figure 2:**
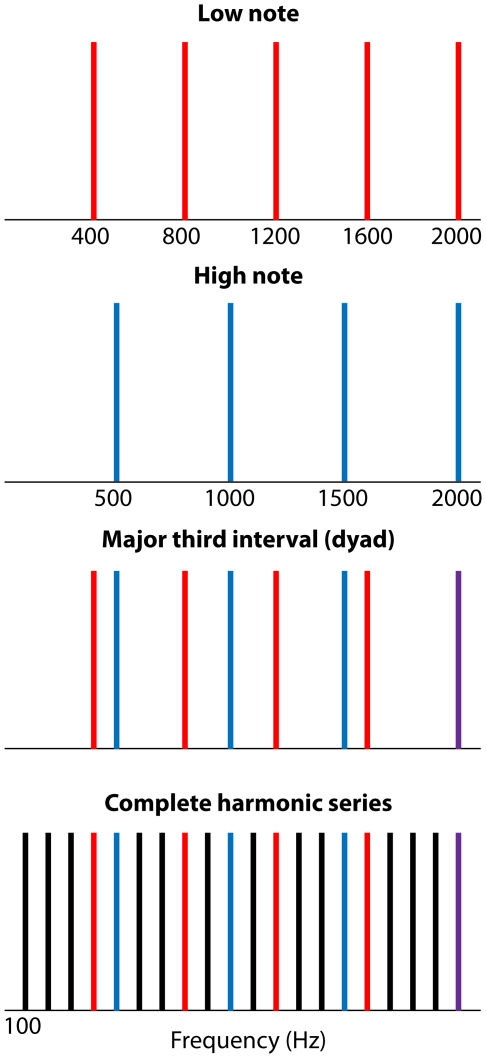
The harmonic structure of a tonal dyad (a major third in this example) compared to a harmonic series. The fundamental frequency of the harmonic series used for comparison with the dyad is given by the greatest common divisor (100 Hz). In this case, the dyad comprises 8 out of the 20 harmonic frequencies in the harmonic series (percentage similarity  = 40%).

The degree of similarity between a two-tone combination (a dyad or interval) and a harmonic series was expressed as the percentage of harmonic frequencies that the dyad held in common with a harmonic series defined by the greatest common divisor of the harmonic frequencies in the dyad (Figure 2). Perceptually, the greatest common divisor of the dyad corresponds to its virtual pitch (or missing fundamental) and is used in much the same way as in algorithms that determine virtual pitch [11], [21]. Since the robustness of a virtual pitch depends on how many of the lower harmonics are present in the stimulus [1], [21], this measure of similarity is both physically and perceptually relevant. For example, a dyad whose spectrum comprises 50% of the harmonic frequencies in a harmonic series would evoke a stronger virtual pitch perception than a dyad with only 10% of these frequencies. We refer to this metric as the *percentage similarity* of a dyad. Percentage similarity can be expressed as ((x+y−1)/(x*y))*100, where x is the numerator of the frequency ratio and y is the denominator of the ratio. For instance, a major third has a frequency ratio of 5∶4; since x = 5 and y = 4, the percentage similarity is 40%.

**Figure 3:**
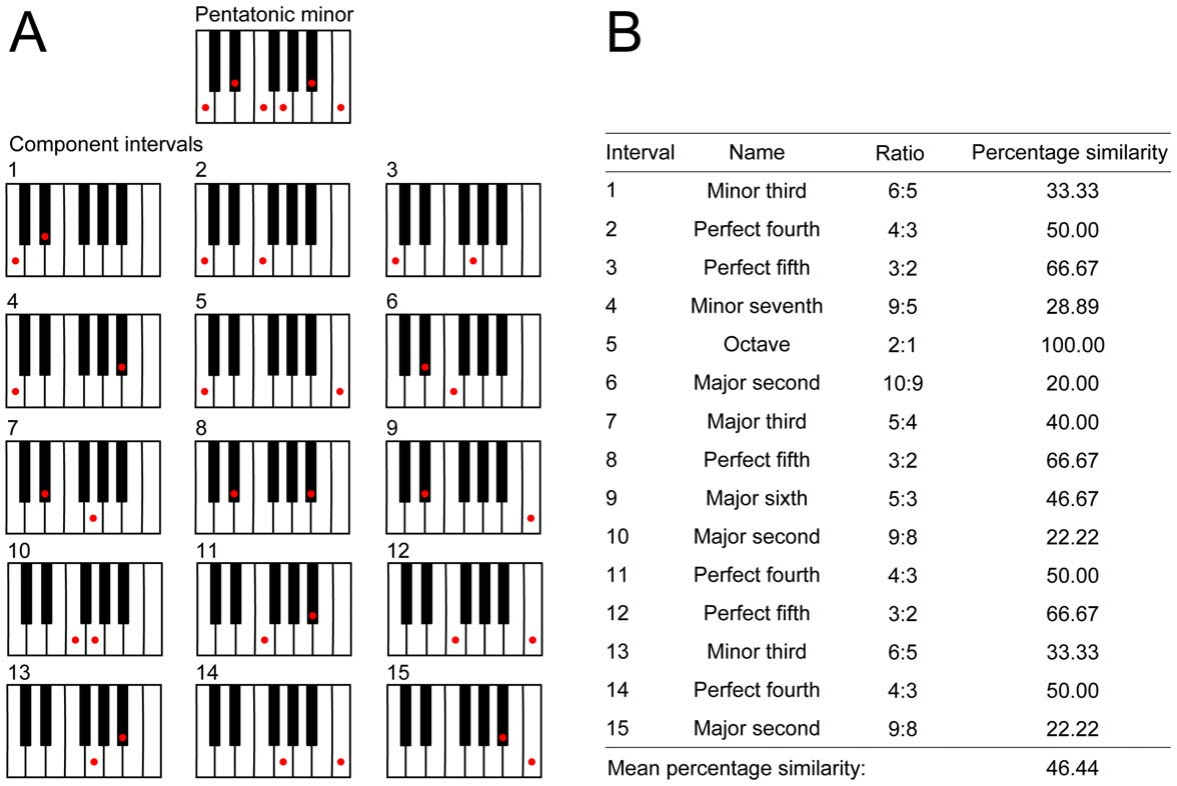
Determination of the mean percentage similarity of a scale, using the pentatonic minor scale as an example. A) The 15 possible intervals between the tones of this scale. B) The percentage similarity of each scalar interval compared to a harmonic series (see Figure 2) and the mean percentage similarity of the full scale are indicated. Scale degrees are conventionally indicated as frequency ratios with respect to a fixed tonic.

The overall conformance of a scale to a harmonic series was then determined by calculating the mean percentage similarity of the dyads in the scale in question (Figure 3). Using the mean as an index of similarity between a scale and a harmonic series implies that all possible dyads in the scale are equally relevant. Although in contemporary Western music any two notes in a scale can, in principle, be used together in melody or harmony, in traditional Western voice-leading and in other musical systems (e.g., classical Indian) particular tone combinations are avoided or prohibited [22]–[24], [25], [26]. Nonetheless, there is no universal rule that describes which intervals might be more important in a scale than others; thus we treated all intervals equally.

Each scale analyzed is bounded by two tonics that are separated by an octave (see Figure 1); thus intervals spanning octaves (e.g., in a natural minor scale, the interval of a major third between the seventh scale degree and the second scale degree in the octave above) are not included in the calculation of the mean percentage similarity. In Western music, intervals spanning octaves are used in melody; however, in particular scales used by other cultures (classical Indian music for example), these intervals are not used [22]–[24]. Given these facts, we do not assume intervals across octaves to be part of any formal scale structure.

Because musical scale tones are not always defined by a single frequency ratio (e.g., the ratios of 7∶5 or 10∶7 can both represent a tritone), the algorithm we used allowed tones within a specific frequency distance to represent the same scale tone. To our knowledge, there is no psychoacoustical data on the size of the frequency window within which intervals are considered musically equivalent. We thus defined the window based on musical practice. Twenty-two cents was used because it is the maximum frequency distance between scale tones that are considered musically equivalent in Western music (i.e., the interval between the minor sevenths defined by ratios of 9∶5 and 16∶9 [7]); it is also the minimum frequency distance between two tones that are considered unique in classical Indian music [3]. Note that 22 cents is significantly larger than the just noticeable frequency difference between tones (around five cents), implying that the size of the window is not based on the resolution of the auditory system. If two or more ratios fell within the 22 cent window, the algorithm defaulted to the ratio yielding the highest percentage similarity from any comparison. For example, if 9∶8 or 10∶9 represented the second scale degree of a scale being tested (these two intervals are within 22 cents of each other), the algorithm would use 9∶8 rather than 10∶9 to form an interval with a perfect fifth (3∶2) because this choice produces the interval (4∶3 versus 27∶20) with the higher percentage similarity. Conversely, the algorithm would use 10∶9 rather than 9∶8 to form an interval with a major sixth (5∶3) because this choice produces the interval (3∶2 versus 40∶27) with the higher percentage similarity.

### Numbers of scales evaluated

The number of scales in any given category that we could have analyzed in theory is given by n!/((n-k)!*k!) where k is the number of different tones in the scale and n the number of discriminable tones over an octave in the middle range of human hearing. If we had considered every discriminable interval over an octave as a potential scale tone, the number of possible scales would have been computationally overwhelming. For example, using the value of 240 discriminable tones over an octave given by Zwicker and Fastl [1], the number of possible seven-note combinations is >10^11^. As a compromise between evaluating as many scales as possible while limiting the computational load, we restricted the potential scale tones to 60 tones (i.e., 25% of the number of discriminable tones in an octave; see Table 1). The 60 tones used were those that, as dyadic combinations with a fixed tonic, had the greatest percentage similarity to a harmonic series. The tones in this subset were separated by 20 cents on average, which is much closer than the ∼100 cent minimum separation of tones in most scales; even classical Indian microtones (srutis) are never separated by less than 22 cents [3]. This restriction left for analysis 455,126 possible pentatonic scales, 45,057,474 heptatonic scales and 279,871,768,995 dodecatonic (12-note) scales (again for reasons of computational efficiency, we analyzed a random sample of only 10 million possible dodecatonic scales). The numbers of possible scales we analyzed are given by n = 59 and k = 4, 6, and 11; 59 was used rather than 60 because the octave is assumed as a component interval of all scales, and 4, 6 and 11 were used rather than 5, 7, and 12 because we treated the first note as a fixed reference point (i.e. a tonic). Thus the tonic note and the octave above it bounded all the scales analyzed. A MATLAB (The Mathworks Inc., Natick MA) algorithm was written to compute the mean percentage similarity for each potential scale and to rank the scales in descending order according to their mean percentage similarity.

**Table 1:** The 60 intervals with the greatest percentage similarity to a harmonic series.

Frequency ratio	Interval size (cents)	Percentage similarity	Frequency ratio	Interval size (cents)	Percentage similarity
2∶1	1200.00	100.00	17∶9	1101.05	16.34
3∶2	701.96	66.67	13∶11	289.21	16.08
4∶3	498.04	50.00	14∶11	417.51	15.58
5∶3	884.36	46.67	13∶12	138.57	15.38
5∶4	386.31	40.00	17∶10	918.64	15.29
7∶4	968.83	35.71	15∶11	536.95	15.15
6∶5	315.64	33.33	16∶11	648.68	14.77
7∶5	582.51	31.43	19∶10	1111.20	14.74
8∶5	813.69	30.00	17∶11	753.64	14.44
9∶5	1017.60	28.89	14∶13	128.30	14.29
7∶6	266.87	28.57	18∶11	852.59	14.14
8∶7	231.17	25.00	19∶11	946.20	13.88
11∶6	1049.36	24.24	15∶13	247.74	13.85
9∶7	435.08	23.81	17∶12	603.00	13.73
10∶7	617.49	22.86	20∶11	1035.00	13.64
9∶8	203.91	22.22	16∶13	359.47	13.46
11∶7	782.49	22.08	21∶11	1119.46	13.42
12∶7	933.13	21.43	15∶14	119.44	13.33
13∶7	1071.70	20.88	19∶12	795.56	13.16
11∶8	551.32	20.45	17∶13	464.43	13.12
10∶9	182.40	20.00	18∶13	563.38	12.82
13∶8	840.53	19.23	17∶14	336.13	12.61
11∶9	347.41	19.19	19∶13	656.99	12.55
15∶8	1088.27	18.33	16∶15	111.73	12.50
11∶10	165.00	18.18	23∶12	1126.32	12.32
13∶9	636.62	17.95	20∶13	745.79	12.31
14∶9	764.92	17.46	17∶15	216.69	12.16
13∶10	454.21	16.92	21∶13	830.25	12.09
12∶11	150.64	16.67	19∶14	528.69	12.03
16∶9	996.09	16.67	22∶13	910.79	11.89

Interval size is the distance from a fixed tonic in cents. See Methods and Figure 2 for further explanation.

The 50 pentatonic and heptatonic scales with the highest mean percentage similarity were individually compared to scales from various cultures including Western, Arabic, Indian, and East Asian. Figure 1 shows the common Western scales used for comparison. These same heptatonic and pentatonic scales constitute most of the basic scale structures of Indian and East Asian music, respectively [3], [9], [10]. The ragas of classical Indian music are particular subsets of tones from these seven-tone “parent” scales or *thats*, and the numbers reported in the literature vary from under one hundred to thousands [3], [22]–[24]. Multiple different sources were used to compile a comprehensive list of over 4000 ragas for comparison with the scales shown in Tables 2 and 3 [op cit.]. Arabic music uses some of the same heptatonic scales shown in Figure 1 (e.g., the Ajam scale is equivalent to the major scale) in addition to uniquely Arabic scales [2], [27]. As with ragas, the numbers of Arabic scales reported vary; two sources were used to compile a list of 35 for comparison [op cit.]. The randomly chosen dodecatonic scales were not individually analyzed, as the chromatic scale is the only musical scale in this category.

**Table 2:** The 50 pentatonic scales whose intervals conform most closely to a harmonic series out of ∼4×10^5^ possibilities examined.

Scale	Scale degrees	Mean percentage similarity	Scale	Scale degrees	Mean percentage similarity
Minor	3∶2, 4∶3, 6∶5, 16∶9	46.44	-----	3∶2, 16∶9, 20∶11, 17∶14	42.61
Ritusen	3∶2, 4∶3, 5∶3, 10∶9	46.44	-----	3∶2, 10∶9, 16∶9, 17∶14	42.59
Candrika todi	3∶2, 4∶3, 6∶5, 8∶5	44.28	-----	3∶2, 4∶3, 7∶4, 15∶13	42.59
Asa-gaudi	3∶2, 4∶3, 5∶3, 5∶4	44.09	-----	3∶2, 4∶3, 17∶10, 15∶13	42.51
-----	3∶2, 4∶3, 9∶8, 22∶13	44.02	-----	4∶3, 5∶3, 9∶5, 17∶14	42.42
Major	3∶2, 5∶3, 5∶4, 10∶9	44.00	-----	3∶2, 4∶3, 10∶9, 17∶15	42.34
Suspended	3∶2, 4∶3, 9∶8, 16∶9	43.95	-----	4∶3, 5∶3, 11∶10, 13∶9	42.34
Man Gong	4∶3, 6∶5, 8∶5, 16∶9	43.85	-----	3∶2, 4∶3, 5∶3, 15∶13	42.34
Catam	3∶2, 4∶3, 5∶3, 6∶5	43.38	-----	3∶2, 5∶3, 5∶4, 15∶8	42.27
-----	4∶3, 5∶3, 9∶5, 11∶10	43.33	-----	4∶3, 5∶3, 9∶8, 17∶10	42.25
-----	3∶2, 4∶3, 10∶9, 17∶14	43.33	-----	4∶3, 5∶3, 9∶8, 17∶12	42.17
-----	3∶2, 4∶3, 15∶14, 21∶13	43.24	-----	3∶2, 5∶4, 10∶9, 15∶8	42.12
-----	3∶2, 4∶3, 17∶14, 17∶15	43.21	-----	3∶2, 5∶3, 9∶8, 17∶10	42.11
-----	3∶2, 4∶3, 5∶3, 9∶5	43.11	-----	3∶2, 5∶3, 10∶9, 20∶11	42.10
-----	3∶2, 4∶3, 15∶14, 19∶12	43.05	-----	4∶3, 5∶3, 9∶8, 16∶9	42.09
-----	3∶2, 4∶3, 17∶15, 21∶13	43.00	-----	3∶2, 4∶3, 16∶9, 21∶13	42.06
-----	3∶2, 4∶3, 5∶3, 17∶15	42.97	-----	4∶3, 8∶5, 16∶9, 15∶14	42.05
-----	3∶2, 4∶3, 16∶9, 15∶14	42.96	-----	3∶2, 4∶3, 5∶4, 17∶15	42.04
-----	4∶3, 5∶3, 9∶8, 11∶10	42.85	-----	3∶2, 4∶3, 15∶8, 17∶15	42.03
-----	4∶3, 5∶3, 5∶4, 10∶9	42.83	-----	3∶2, 5∶3, 9∶8, 17∶12	41.95
-----	3∶2, 8∶5, 16∶9, 17∶14	42.80	-----	3∶2, 4∶3, 10∶9, 19∶12	41.91
-----	3∶2, 6∶5, 10∶9, 20∶11	42.73	-----	3∶2, 5∶3, 10∶9, 15∶8	41.90
-----	3∶2, 5∶3, 10∶9, 17∶14	42.64	-----	3∶2, 8∶5, 20∶11, 17∶14	41.88
-----	3∶2, 4∶3, 10∶9, 20∶11	42.62	-----	3∶2, 4∶3, 5∶4, 10∶9	41.87
-----	3∶2, 5∶4, 15∶8, 17∶15	42.61	-----	3∶2, 4∶3, 16∶9, 19∶12	41.87

Scale degrees are indicated as frequency ratios with respect to a fixed tonic; the ordering of scale degrees is based on decreasing percentage similarity both here and in Table 3.

**Table 3:** The 50 heptatonic scales whose intervals conform most closely to a harmonic series out of ∼4×10^7^ possibilities examined.

Scale	Scale degrees	Mean percentage similarity	Scale	Scale degrees	Mean percentage similarity
Phrygian	3∶2, 4∶3, 6∶5, 8∶5, 16∶9, 15∶14	40.39	-----	3∶2, 4∶3, 16∶9, 15∶13, 17∶14, 21∶13	38.03
Dorian	3∶2, 4∶3, 5∶3, 6∶5, 9∶5, 10∶9	39.99	-----	3∶2, 4∶3, 20∶11, 15∶14, 17∶14, 21∶13	38.01
Major	3∶2, 4∶3, 5∶3, 5∶4, 10∶9, 15∶8	39.61	-----	3∶2, 4∶3, 5∶3, 16∶9, 17∶14, 21∶13	37.97
Husayni	3∶2, 4∶3, 6∶5, 8∶5, 9∶5, 12∶11	39.39	-----	3∶2, 4∶3, 16∶9, 15∶14, 19∶12, 17∶15	37.97
Natural minor	3∶2, 4∶3, 6∶5, 8∶5, 9∶8, 16∶9	39.34	-----	3∶2, 4∶3, 5∶3, 5∶4, 9∶8, 17∶10	37.95
Lydian	3∶2, 5∶3, 5∶4, 10∶7, 9∶8, 15∶8	38.95	-----	3∶2, 4∶3, 5∶3, 9∶8, 15∶8, 17∶12	37.95
-----	3∶2, 4∶3, 6∶5, 9∶8, 16∶9, 17∶10	38.83	-----	3∶2, 4∶3, 5∶3, 9∶5, 15∶14, 17∶14	37.95
Kardaniya	3∶2, 4∶3, 5∶3, 11∶6, 10∶9, 17∶14	38.76	-----	3∶2, 4∶3, 5∶3, 16∶9, 19∶12, 17∶14	37.94
-----	3∶2, 4∶3, 5∶3, 9∶8, 17∶10, 15∶13	38.69	-----	3∶2, 4∶3, 16∶9, 15∶13, 19∶12, 17∶14	37.93
Mixolydian	3∶2, 4∶3, 5∶3, 5∶4, 9∶5, 9∶8	38.59	-----	3∶2, 4∶3, 5∶3, 9∶5, 11∶10, 13∶9	37.92
-----	3∶2, 4∶3, 10∶9, 20∶11, 17∶14, 21∶13	38.39	-----	3∶2, 4∶3, 5∶3, 9∶5, 9∶8, 15∶14	37.91
-----	3∶2, 4∶3, 20∶11, 19∶12, 17∶14, 17∶15	38.33	-----	3∶2, 4∶3, 16∶9, 17∶12, 17∶14, 17∶15	37.90
-----	3∶2, 4∶3, 5∶3, 9∶8, 17∶10, 17∶14	38.30	-----	3∶2, 4∶3, 8∶5, 12∶7, 17∶14, 17∶14	37.89
-----	3∶2, 4∶3, 5∶3, 10∶9, 19∶12, 17∶14	38.23	-----	4∶3, 5∶3, 9∶5, 11∶10, 19∶12, 17∶14	37.87
-----	3∶2, 4∶3, 20∶11, 17∶14, 17∶15, 21∶13	38.21	-----	3∶2, 4∶3, 5∶3, 9∶5, 9∶8, 17∶10	37.85
-----	3∶2, 4∶3, 16∶9, 15∶14, 17∶14, 17∶15	38.19	-----	3∶2, 4∶3, 9∶8, 17∶10, 20∶11, 17∶14	37.85
-----	3∶2, 4∶3, 5∶3, 5∶4, 9∶8, 17∶12	38.14	-----	3∶2, 4∶3, 5∶3, 9∶5, 9∶8, 17∶12	37.85
-----	3∶2, 4∶3, 20∶11, 15∶14, 19∶12, 17∶14	38.13	-----	3∶2, 4∶3, 5∶3, 9∶8, 17∶10, 17∶12	37.83
-----	3∶2, 4∶3, 10∶9, 16∶9, 15∶14, 17∶14	38.11	-----	3∶2, 4∶3, 15∶14, 17∶14, 17∶15, 21∶13	37.79
-----	3∶2, 4∶3, 5∶3, 10∶9, 17∶14, 21∶13	38.09	-----	3∶2, 4∶3, 9∶7, 16∶9, 17∶14, 21∶13	37.78
-----	3∶2, 4∶3, 5∶3, 5∶4, 9∶8, 17∶14	38.07	-----	4∶3, 5∶3, 9∶5, 11∶10, 13∶9, 17∶14	37.77
-----	3∶2, 4∶3, 16∶9, 15∶14, 17∶15, 21∶13	38.07	-----	3∶2, 4∶3, 5∶3, 9∶8, 17∶12, 17∶14	37.74
-----	3∶2, 4∶3, 5∶3, 5∶4, 9∶5, 11∶10	38.05	-----	3∶2, 4∶3, 5∶3, 16∶9, 17∶14, 17∶15	37.74
-----	3∶2, 4∶3, 16∶9, 17∶12, 15∶14, 17∶14	38.04	-----	3∶2, 4∶3, 15∶14, 19∶12, 17∶14, 17∶15	37.69
-----	3∶2, 4∶3, 10∶9, 20∶11, 18∶13, 17∶14	38.04	Locrian	4∶3, 6∶5, 8∶5, 10∶7, 16∶9, 15∶14,	37.68

### The use of justly tuned intervals

Western music over the last few centuries has been based on equal temperament tuning, which developed as a compromise between the aesthetic value of maintaining justly tuned intervals (i.e., intervals defined by relatively small integer ratios) and the practical need to facilitate musical composition and performance in multiple keys, especially on keyboard instruments [28], [29]. Just intonation is generally considered the most natural tuning system and was the system used before orchestras, composers and instrument makers demanded equal temperament (op cit.). Moreover, just intonation is used in non-Western traditions such as classical Indian music [3], [22]–[24]. The scales analyzed in the present study are therefore justly tuned.

## Results

### Pentatonic scales

Table 2 lists the 50 five-note scales among the >4×10^5^ possibilities evaluated in this category with the highest mean percentage similarity to a harmonic series. The scale topping the list is the minor pentatonic scale, one of the most widely used five-note scales [5]. The second highest ranked is the Ritusen scale, a pentatonic mode used in traditional Chinese and Indian music (see Figure 1; [3], [9], [10], [22]–[24]). The third and fourth ranked pentatonic scales are the ascending forms of two ragas (Candrika todi and Asa-gaudi) used in classical Indian music [3]. Although these two scales are not formally recognized in Western music theory, they can be thought of as the natural minor and major heptatonic scales, respectively, with the second and seventh scale degrees excluded. Thus some Western melodies are likely to use these particular combinations of tones. The fifth ranked pentatonic scale is identical to the Ritusen scale (known as the Durga raga in classical Indian music) except that the fifth scale degree (17∶10 in this case) is ∼34 cents sharp (i.e., higher in frequency) compared to the 5∶3 major sixth in the Ritusen scale. Because a sharp sixth interval is musically acceptable in certain contexts in classical Indian music, this scale may indeed represent the Durga raga (see Discussion). The sixth through eighth ranked five-note scales are the remaining modes of the major/minor pentatonic scale (see Figure 1), and the ninth ranked scale is the Catam raga [3].

### Heptatonic scales

The 50 heptatonic scales with the highest mean percentage similarity among the >4×10^7^ possible scales evaluated are shown in Table 3. Three of the seven heptatonic modes (see Figure 1) emerge at the top of this list. The Phrygian mode holds the highest rank followed by the Dorian mode and the Ionian mode (the major scale). The fourth ranked scale is similar to the Phrygian mode but contains a neutral second (12∶11) instead of a minor second; this collection is the Husayni scale in Arabic music [27]. The Aeolian mode (the natural minor scale) and Lydian mode are the fifth and sixth ranked scales. The next three scales are similar to the Dorian mode but with slight alterations in one or two scale degrees. The seventh ranked scale may represent the Kafi scale in classical Indian music with an alternative sharp sixth scale degree [22]. The eighth ranked scale is the Kardaniya scale in Arabic music [op cit.]. Although the ninth ranked scale does not represent any well-known musical tone collection, the Mixolydian mode is ranked tenth. The Locrian, which is the least used of the Western modes, is ranked fiftieth. Thus both the five-note and seven-note scales preferred in much music worldwide comprise intervals that conform optimally to a harmonic series.

### Dodecatonic scales

A further question is the status of the chromatic scale, which divides octaves into 12 approximately equal intervals (semitones). Both Western and Chinese music theory use the chromatic scale as an organizing principle.

When we compared the chromatic scale to a random sample of 10 million other possible 12-note scales, we found that ∼1.5 million had higher mean percentage similarity to a harmonic series, and none of these, to our knowledge, have been used in music. These results are in sharp contrast to the commonly used five- and seven-note scales that rank at or near the top of their respective groupings. This observation suggests that the chromatic scale has no basis in similarity to a harmonic series. This result is consistent with the fact that the full set of 12 tones is not as widely used as the five-and seven-tone subsets shown in Figure 1, and is considered by some to be less accessible to listeners [14], [30]. Nonetheless, modern composers such as Schoenberg, Webern and Berg have used the chromatic scale as a basis for musical compositions.

## Discussion

The results we report indicate that musical scale preferences are predicted by the overall similarity of their component intervals to a harmonic series. However, several caveats and the possible reasons behind this preference deserve mention.

### Competing explanations of interval preference

Although the results shown in Tables 2 and 3 suggest that musical intervals that are maximally similar to a harmonic series are favored, a number of other explanations of interval preferences have been proposed over the years. One historically important theory was suggested by Helmholtz [19], who argued that dissonant musical tone combinations are produced by inadequate harmonic overlap. In other words, when the harmonics of two musical tones fall within the minimum frequency distance at which two pure tones can be individually resolved by humans (the critical bandwidth), an unpleasant perception of “beating and roughness” occurs (see also refs. 11, 39–42). Another explanation for interval preferences is based on the relationships among the harmonics produced by the voice or by musical instruments [20], [21]. In this view, the frequency ratios between lower, more powerful harmonics are more readily appreciated, leading to a perceptual preference for dyads whose fundamentals are smaller integer ratios. A third interpretation of scale preferences is based on the elicitation of more harmonious virtual pitches [45]. For example, in addition to the perception of the pitches of the two component tones, a perfect fifth elicits the perception of a virtual pitch an octave below the lower tone. In this theory, such virtual pitches could make an interval more consonant.

Whether any of these theories of dyadic preference could account for scale preferences in music has not been examined. Nonetheless, the rankings of interval preferences predicted by these theories are similar to one another and to the ranking predicted by harmonic series-similarity (see Table 1) [19]–[21], [39]–[42], [45]. This is not surprising since each theory was developed to explain the same generally accepted consonance ranking of dyads. Thus any of these theories could account for scale preferences if the metrics were quantified and used in the algorithm presented here in place of percentage similarity. For example, the scales with the highest mean percentage similarity are likely to be the ones with the highest mean harmonic overlap or lowest mean beating. It is impossible to tease apart the metric or combination of metrics that is responsible for scale preferences using this algorithm alone. It is noteworthy, however, that these theories are all variations of the same general idea, namely a human preference for particular characteristics of harmonic series.

### A biological rationale

Why then should this preference exist? Although other explanations cannot be ruled out based on the data we have presented, for the reasons discussed in this section, we favor a biologically based preference for harmonic series as the most plausible explanation for the particular scales used to make music over history and across cultures.

Like any other sensory quality, the human ability to perceive tonal (i.e., periodically repeating) sound stimuli has presumably evolved because of its biological utility. In nature, such sound stimuli typically occur as harmonic series produced by objects that resonate when acted on by a force [19], [31]. Such resonances occur when, for example, wind or water forces air through a blowhole or some other accidental configuration, but are most commonly produced by animal species that have evolved to produce periodic sounds for social communication and ultimately reproductive success (e.g., the sounds of stridulating insects, the vibrations produced by the songbird syrinx, and the vocalizations of many mammals). Although all these harmonic stimuli are present in the human auditory environment, the vocalizations of other humans are presumably the most biologically relevant and frequently experienced.

In humans, vocal stimuli arise in a variety of complex ways, not all of which are harmonic. Harmonic series depend on vocal fold vibrations and are characteristic of the “voiced speech” responsible for vowel sounds and some consonants [1]. Although the relative amplitudes of harmonics are altered by filtering effects of the supralaryngeal vocal tract resonances to produce different vowel phones, the frequencies of harmonics remain unchanged [op cit.]. In consequence, the presence of a harmonic series is a salient feature of human vocalizations and essential to human speech and language. It follows that the similarity of musical intervals to harmonic series provides a plausible biological basis for the worldwide human preference for a relatively small number of musical scales defined by their overall similarity to a harmonic series.

Several lines of evidence accord with this idea. First, humans and other primate species are specifically attracted to conspecific vocalizations, including those with harmonic and even specifically musical characteristics [32]–[38]. Second, the human pinna, ear canal and basilar membrane are all optimized to transmit human vocalizations, suggesting that the human sense of tonality co-evolved to respond to the stimuli generated by the vocal tract [31], [39], [40]. Third, a number of non-musical phenomena in tone perception including perception of the missing fundamental, pitch shift of the residue, spectral dominance and pitch strength can be explained by in terms of the specialization of the human auditory systems for processing vocal sounds [41]–[43]. These observations all support the idea that the musical scales used over human history have resulted from a preference for collections of dyads that most resemble a harmonic series, and therefore human vocalizations.

### The biological relevance of other musical features

This interpretation raises the question of whether other features of human vocalizations are, for similar reasons, influential in musical preferences. In addition to harmonicity per se, particular frequency ranges, timbres and prosodic fluctuations make vocalizations specifically human and may be equally or more influential in musical preferences. In support of this idea, non-human primates have been recently shown to respond affectively to music characterized by frequency ranges and prosody that are similar to their own vocalizations [44]. This evidence accords with the fact that most music, even purely instrumental music, is composed within the human vocal range, and some popular instruments (e.g., the violin) bear a timbral resemblance to the human voice [31]. Moreover, many musical traditions use tones that fall between formal scale tones: in Western music, glissandos involve continuous changes in pitch, blues music depends on “bending” guitar strings to blend the pitches of major and minor thirds, and classical Indian music employs microtonal intervals that fall between the scale tones of ragas [24]. These musical embellishments may reflect the continuous variations in fundamental frequencies that characterize speech prosody. Preferred meters and tempos may also parallel speech and other vocalizations in ways that do not involve tonality at all [6]. Thus while scale preferences seem to be based on the harmonic series that derive from vocal fold vibrations, other aspects of music may be favored because they resemble additional features of the human voice.

### The different usage of highly ranked scales

Although many of the highly ranked heptatonic and pentatonic scales in Tables 2 and 3 have been widely used in Western music, some others have not. A possible explanation is that whereas all the modes shown in Figure 1 can be played using the same set of intervals, one or more additional intervals (e.g., a neutral second) would be necessary to play the other highly ranked but little used variations. Since it is relatively easy to play the modes on the same instrument with the same tuning, this property has both practical and theoretical appeal. Nonetheless, some of these other scales are used in non-Western cultures [3], [23], perhaps because their instrumentation favors non-Western tonal relationships. A few highly ranked scales in Tables 2 and 3 may not be used in music simply because they differ so little from the scales that are used. For example, the ninth ranked heptatonic scale is not, to our knowledge, recognized as a scale in its own right. It is, however, nearly the same as the Durga raga with microtonal embellishments, as well as to the Kafi scale and the Dorian mode, both of which have a higher mean percentage similarity.

A related concern is why the ordering of the widely used five-note and seven-note scales from greatest to least mean percentage similarity values in Tables 2 and 3 does not simply follow the order of their popularity, at least in Western music. For example, the major and natural minor heptatonic scales prevalent in Western music today rank below the Phrygian and Dorian modes. One possibility is again instrumentation. For instance, an early explanation for using the Aeolian mode as opposed to another minor mode (e.g., Dorian) was to facilitate performance on particular instruments in certain tunings [46].

A scale that deserves special comment is the Locrian mode, which ranks much lower in Table 3 than the other modes. The Locrian mode is recognized in Western music theory but rarely used. While reasons cited for the infrequent use of the Locrian mode are its weak tonal center and dissonant tonic chord, it may be less desirable primarily because of the relatively low conformance of its intervals to a harmonic series and thus to the biological signature of voiced speech and other harmonic vocalizations.

Finally, although many of the widely used scales in music worldwide hold high ranks in Tables 2 and 3, scales that are used in the music of a few cultures do not. For example, the sléndro scale used in Javanese gamelan music comprises five approximately equally spaced tones over an octave [47], [48] but is not among the pentatonic scales with the highest mean percentage similarity to a harmonic series. A possible explanation in this case is that the metallophone instruments used by gamelan orchestras (e.g., bells and gongs that are idiosyncratic to a given geographical region) generate non-harmonic frequencies. Thus the present analysis based on harmonic series is not applicable to such instruments or the scales that derive from them. It should also be noted that several Arabic scales examined are not present in Table 3. One possible explanation is that the most commonly used scales are those in Table 3, while the less commonly used scales have lower percentage similarity. However, to our knowledge, there is no consensus about which Arabic scales are most frequently used to make music. Alternatively, harmonic series similarity may not the only factor influencing scale preferences in this culture. By the same token, only a few of the hundreds to thousands of classical Indian ragas are represented among the highly ranked pentatonic and heptatonic scales. However, nearly all the “parent” scales (*thats*) from which all ragas are derived are among the highly ranked heptatonic scales indicated by their Western names in Figure 1 and Table 3.

### The relative popularity of five- and seven-tone scales

The fact that most musical scales emphasize five or seven tones raises the question of why such scales are preferred over those with a larger or smaller numbers of tones. As the number of tones in a scale decreases, the similarity of the tone collection to the character of a harmonic series increases (compare the percentage similarity values of the top-ranked pentatonic and heptatonic scales in Tables 2 and 3). Conversely, dividing octaves into a larger number of intervals leads to tonal collections that meet this criterion less well. Thus under the hypothesis that listeners prefer tone collections whose spectra are on average more like a harmonic series, the inclusion of intervals that conform to this criterion relatively poorly would provide an upper bound on the number of preferred scale tones.

Since tone collections with fewer notes have greater similarity to a harmonic series, it is less clear why tone collections smaller than five notes are not preferred. One reason may be that as the number of scale tones that divide an octave decreases, the distance between successive notes necessarily increases. Larger intervals are more difficult to sing [5], presumably because the associated changes in vocal fold tension and vocal tract shape require a greater expenditure of neuromuscular energy and practice to develop the necessary coordination. Multiple sequential skips (intervals of a third or greater) are discouraged in traditional rules of voice-leading for this reason [25], [26]. Thus beyond a relatively small number of scale tones (e.g., five), a further decrease would increase the difficulty of vocal (or instrumental) performance, outweighing the gain in harmonic series similarity. Moreover, decreasing the number of scale tones decreases the variety of intervals available for musical composition. In short, the number of tones used in popular scales may be a compromise between these competing factors.

A further issue is the place of six-note scales, which seem less frequently used than five- or seven-note scales. In fact, blues scales, which are prevalent in popular music today, are often classified as six-note variants of five- or seven-note scales and considered hexatonic scales by some musicologists [49]–[51]. Six tones are also used in particular Indian ragas [3], [22]–[24]. Melodies using heptatonic scales sometimes use only six out of the seven tones, and melodies using pentatonic scales often use passing tones not included in the scale structure as such [5]. Such compositions could also be interpreted as using six-note scales. Thus there is certainly nothing prohibitive about using a set of six tones to create music; they are simply not recognized as formally as their five- and seven-note counterparts in Western music theory.

### The method of analyzing scales

The algorithm we used to analyze scales is unique in that it accounts for every possible interval between scale tones over an octave. Other analyses have focused on intervals between tones and the tonic [15], [16], [20]. Accounting for all possible intervals is essential to our argument and essential to understanding the historical fact that intervals between any two scale tones can be heard as consonant or dissonant and affect the overall appeal of the scale [28], [29]. An algorithm of this sort has the further virtues of being able to incorporate other metrics of interval comparison (see above) and of demonstrating the spectral similarity of the scales commonly used in Western, Indian, Chinese and Arabic music (see Tables 2 and 3).

### Conclusions

The analyses we report here show that many of the relatively small number of scales that humans have preferred over history and across cultures comprise intervals that when considered as a set are maximally similar to harmonic series. The basis for these results may be a preference for the biologically significant spectral features that characterize conspecific vocalizations.
